# Understanding the cataract treatment disparities among older adults in India

**DOI:** 10.3389/fpubh.2024.1424031

**Published:** 2024-08-05

**Authors:** Rajeev Ranjan Singh, Sanjay K. Mohanty

**Affiliations:** ^1^Research Scholar, International Institute for Population Sciences, Mumbai, India; ^2^Department of Population & Development, International Institute for Population Sciences, Mumbai, India

**Keywords:** visual impairments, cataract, ageing, Lasi, India

## Abstract

**Background:**

Cataract is a leading cause of global blindness, affecting around 33% of blind individuals worldwide. It significantly impacts individuals’ well-being, independence, and quality of life, posing a substantial economic burden. India’s rapidly ageing population necessitates an examination of cataract prevalence and treatment disparities. No attempts have been made to address socioeconomic variation in treatment disparities of effective cataract treatment coverage among older adults in India.

**Data and method:**

This study utilises data from the Longitudinal Ageing Study of India (LASI) conducted in 2017–18, that covered, 73,396 individuals aged 45 and above. Logistic regression, univariate, and bivariate analyses were employed to understand the variation of cataract and their associations with various demographic factors. Visual acuity tests and self-reported cataract data were used.

**Results:**

The prevalence of cataract among older adults in India was 14.25%, with higher rates among females and the older adult. Socioeconomic disparities werelarge, with lower prevalence among those with higher education and urban residence. Despite the effectiveness of cataract surgery, disparities in treatment access and effective coverage persisted. Approximately 27.52% of older adults did not receive cataract treatment, and those who received out of them 28% did not receive effective treatment. The effective treatment was lower among female, less educated, and poor.

**Conclusion:**

Cataract remains a significant public health concern in India, particularly among older adults. The study highlights the importance of addressing socioeconomic disparities in cataract treatment access and quality of care. Targeted interventions are needed to bridge these gaps, ultimately improving visual health outcomes and well-being among older adults in India.

## Introduction

Cataract is the leading cause of visual impairment and blindness worldwide, accounting for one third of global blindness (1–4). Additionally, cataract is one of the major reasons for moderate and severe visual impairment (5). This condition is characterised by the clouding of the lens, resulting in a gradual deterioration of vision. The impact of cataract extends beyond visual impairment, as it can significantly affect individuals’ overall well-being, independence, and quality of life, imposing a substantial economic burden on individuals and society (6, 7). Cataract and uncorrected refractive error are the two major contributors of global visual impairment. About 80% of all visual impairments can be avoided or treated by addressing these two. While a pair can cure refractive errors of suitable glasses or lenses, cataract can be removed through surgery only. It is a common age-related eye condition that significantly burdens the global population, particularly among older adults (8, 9).

The demographic transition and improved healthcare access have led to a growing number of older adults and older adult individuals in India (10, 11). The older adult population is growing about 3 times higher than the overall population in India and is likely to be 20% of the population share by the year 2050 (12). Consequently, there is a pressing need to understand the age-related health challenges faced by this population segment, with cataract being a critical area of concern. India faces a significant burden of age-related cataracts. Although cataract surgery coverage is growing, the unaddressed demand for such surgeries remains substantial (13). Though India launched a centrally sponsored National Programme for Control of Blindness (NPCB) in the year 1976, and it has greatly improved cataract surgery coverage. It requires further strengthening to effectively reach the most deprived segments of society. Furthermore, with increasing longevity, the age at onset of Non-Communicable Diseases (NCDs) is also decreasing (14, 15), specially diabetes as the likelihood of visual impediment is higher among diabetic patients (16). Cataracts cause vision loss without early warning signs or symptoms, and by the time symptoms appear, the disease has often advanced significantly. Several studies have explored the prevalence and risk factors of cataract in India (13, 16–19).

Although cataract surgery is a highly effective intervention, evidence suggests disparities in the accessibility and utilization of cataract treatment services in India (20, 21). Existing studies have indicated that factors such as income, education, and residence influence the likelihood of cataract surgery (22, 23). Comprehensive analysis of these socioeconomic disparities of cataract treatment coverage and effective cataract treatment is lacking in India. There is limited research on the barriers disadvantaged groups face in accessing cataract treatment services.

This paper seeks to contribute knowledge on cataract in India by estimating the prevalence, disparities in cataract treatment, and effective treatment coverage among older adults in India. The study shows the magnitude of the problem and provide evidence-based insights for policy formulation and healthcare interventions. Furthermore, the results may inform strategies for early detection, prevention, and treatment of cataract, ultimately leading to improved visual health outcomes and an enhanced quality of life for older adults in India.

## Data and method

### Data

The study utilizes data from the first wave of the Longitudinal Ageing Study of India (LASI), conducted in 2017–18. LASI data was collected with a collaboration between the International Institute for Population Sciences (IIPS), the University of Southern California (USC), and the Harvard T.H. Chan School of Public Health (HSPH). The LASI framework is aligned with other global ageing studies such as the Survey of Health, Ageing, and Retirement in Europe (SHARE), the Health and Retirement Survey (HRS) from the United States, the Korean Longitudinal Study of Aging (KLoSA), and the China Health and Retirement Longitudinal Study (CHARLS). The survey successfully interviewed 73,396 individuals aged 45 and above, along with their spouses of any age, covering all states and union territories in India. Its primary objective was to gain insights into the social, economic, and health aspects of older adults (45+) in India. Prior informed consent from all respondents was obtained. The LASI includes a module on biomarkers and direct health examinations of individuals. The survey was carried out using a multistage stratified area probability sampling approach, making its estimates applicable at both state and national levels. Within each state, a three-stage sampling design was used in rural areas, and a four-stage design was used in urban areas. In rural areas, the first stage involved selecting the primary sampling unit (sub-districts; Tehsil/Taluka), followed by villages in the second stage, and households in the third stage. In urban areas, the process was similar: the primary sampling unit (sub-districts; Tehsil/Taluka) was selected first, followed by wards in the second stage, census enumeration blocks (CEBs) in the third stage, and households in the fourth and final stage. The survey thoroughly documented the socioeconomic status of households and included various biomarkers such as blood pressure, lung function, visual acuity, anthropometry, and grip strength. Visual acuity testing was conducted following World Health Organization (WHO) guidelines, using computer-assisted personal interviews (CAPI) and the tumbling E log medicine administration record (MAR) chart, or log mart vision chart, to assess visual impairment and refractive errors (24). Detailed information about the sampling methodology and survey results can be accessed publicly (24). The effective sample size for this study consisted of 66,606 older adults aged 45 years and above.

### Statistical analysis

The analysis focused on older adults and the older adult, specifically those aged 45 and above, excluding their younger spouses. Preliminary analysis involved the use of descriptive statistics and bivariate analysis. The Chi-square test was employed to examine associations and determine significance levels. Additionally, binary logistic regression was conducted to assess the covariates of cataract among older adults and the older adult in India.

### Outcome variable

The self-reported cataract condition was coded as ‘1’ if Yes and ‘0’ if No. We used blindness, distance vision loss, and near vision loss to define visual impairments, which were further used to segregate effectively controlled cataract and *vice-versa*. An individual was classified as visually impaired if she/he has any or more than one of these impairments. Low distance vision was characterized by visual acuity between 20/80 and 20/200 in the better eye with the best correction available. Low near vision was defined as visual acuity between 20/80 and 20/400 in the better eye with the best correction available. Blindness was determined by an inability to detect light, count fingers at 2 feet, or having visual acuity poorer than 20/400 for near vision or 20/200 for distance vision in the better eye with the best correction available.

### Independent variable

The study incorporated a range of independent variables in its analysis. These are age group (45–54, 55–64, 65–74, and 75+), sex (male/female), level of education (illiterate, less than 5 years, 5–9 years, and 10 years and above), residential type (rural/urban), religious affiliation (Hindu, Muslim, Christian, and others), marital status (currently married, widowed, others), living arrangements for older adults (living alone, with a spouse and/or others, with a spouse and children, with children and/or others), substance use (Yes/No), possession of health insurance (Yes/No), social grouping (Scheduled Caste, Scheduled Tribe, Other Backward Class, and others), and Monthly *Per Capita* Expenditure (MPCE) Quintile. MPCE, which represents the average consumption *per capita* of households, is computed from a series of questions about household expenses on food and non-food items during a specified period. Additionally, adjustments were made to the estimates for age and gender to account for the age-sex distribution found in nationally representative data. In the logistic regression model, the dependent variable was the prevalence of cataract, coded as “1” if present and “0” if absent, while all the independent variables were included in the analysis.

## Results

Table 1 shows the socio-demographic characteristics of the sample population. The average age of the sample population was 60 years, and the mean years of schooling was 8 years. 31% were urban residents, and around 28% belonged to the socially deprived class (SC/ST). about 27% of the population had at least one chronic disease, and 18% have two and above chronic conditions. The prevalence of cataract was 14.25% (15.38% among females and 12.92% among males). Further, 13% of the population were suffering from distance vision loss, 32% from near vision loss, 1.63% from blindness, and around 36% from any visual impairments.

**Table 1:** Socio-demographic characteristics of sample population, India.

Mean age (in years)	60.32 (60.24–60.40)
Mean years of schooling (in years)	8.00 (7.96–8.04)
Percent urban	31.47 (30.42–32.53)
Percent SC/ST	27.76 (27.10–28.43)
Percent Working	62.45 (61.51–63.38)
**Number of chronic diseases**		
0	54.02 (53.13–54.91)
1	27.49 (26.81–28.18)
2+	18.50 (17.62–19.41)
**Cataract**	14.25 (13.75–14.77)
Male	12.92 (12.27–13.59)
Female	15.38 (14.64–16.15)
Distance vision loss	12.77 (12.27–13.30)
Near vision loss	31.62 (30.85–32.41)
Blindness	1.63 (1.44–1.84)
Any visual impairment*	35.93 (34.82–36.21)
**Total (N)**	66,606

*Any visual impairment includes either or all of the distance vision loss/near vision loss/blindness.

Table 2 shows the prevalence of cataract by socioeconomic variables among older adults. The prevalence of cataract was 2.1% higher among females (13.22%) than males (11.12%). By level of education, the cataract was higher among illiterate females (14%) than the male (12.97%)—prevalence of cataract increases with age. In the age group 45–54, it was 4% among females and 2.4% among males; in the age group 75 and above, the prevalence increased to 30.49% among females and 27.97% among males. The prevalence of cataracts was higher among females (16.31%) than males (12.88%) in urban areas compared to rural resident females (11.51%) and males (10.2%). In the case of social class, except for STs (females 6.72% and males 5.64%), the prevalence was high among all other social groups. Similarly, the prevalence was high by religion except for the Christians (female 6.55% and male 5.28). By living arrangement, the prevalence was very high among females (20.84%) living alone compared to males (12.73%) living alone; the prevalence was lowest among those living with a spouse or children (female 8.75% and male 9.35%). The prevalence of cataract increases with the increasing number of chronic conditions. For instance, the prevalence was low among those with no chronic condition (females 9.33% and males 7.82%) compared to those with chronic condition two and above (females 21.82% and males 19.81%).

**Table 2:** Prevalence of cataract by socioeconomic variables among older adults in India.

Background variables	Female	Male	Difference (F–M)
**Sex**	13.22 (12.86 13.57)	11.12 (10.77 11.47)	2.1*** (1.6 2.59)
**Age group**			
45–54	4.01 (3.68 4.35)	2.4 (2.12 2.69)	1.61*** (1.17 2.05)
55–64	11.6 (11.01 12.2)	8.86 (8.28 9.44)	2.75*** (1.91 3.58)
65–74	23.93 (22.96 24.9)	19.63 (18.72 20.54)	4.3*** (2.97 5.63)
75+	30.49 (28.98 32.01)	27.97 (26.44 29.5)	2.53*** (0.37 4.68)
**MPCE quintile**			
Poorest	13.19 (12.41 13.98)	11.12 (10.33 11.92)	2.07*** (0.95 3.19)
Poorer	12.57 (11.8 13.33)	11.06 (10.28 11.84)	1.51*** (0.41 2.6)
Middle	13.4 (12.61 14.19)	10.8 (10.03 11.58)	2.6*** (1.49 3.7)
Richer	14.07 (13.26 14.88)	11.22 (10.44 12)	2.85*** (1.72 3.97)
Richest	12.86 (12.07 13.64)	11.39 (10.6 12.18)	1.47*** (0.35 2.58)
**Educational attainment**			
Illiterate	13.69 (13.23 14.15)	10.5 (9.89 11.11)	3.19*** (2.43 3.96)
Less than 5 years	14.03 (12.87 15.19)	12.97 (11.94 13.99)	1.07*** (−0.48 2.61)
5–9 years completed	12.92 (12.1 13.75)	11.01 (10.35 11.67)	1.91*** (0.86 2.97)
10 years or more	10.47 (9.53 11.41)	11.04 (10.37 11.71)	−0.57*** (−1.72 0.58)
**Residence**			
Rural	11.51 (11.09 11.92)	10.2 (9.78 10.61)	1.31*** (0.73 1.9)
Urban	16.31 (15.67 16.96)	12.88 (12.24 13.52)	3.43*** (2.53 4.34)
**Caste**			
Scheduled Tribes	6.72 (6.1 7.34)	5.64 (5.03 6.25)	1.08*** (0.21 1.95)
Scheduled Castes	13.28 (12.41 14.14)	11.4 (10.52 12.27)	1.88*** (0.65 3.11)
OBC	14.34 (13.74 14.93)	12.21 (11.62 12.8)	2.13*** (1.29 2.96)
Others	15.78 (15.06 16.49)	12.92 (12.21 13.62)	2.86*** (1.86 3.87)
**Religion**			
Hindu	13.95 (13.53 14.37)	11.99 (11.56 12.41)	1.96*** (1.36 2.56)
Muslim	15.8 (14.7 16.9)	11.81 (10.75 12.88)	3.98*** (2.46 5.51)
Christian	6.55 (5.73 7.36)	5.28 (4.49 6.08)	1.26*** (0.13 2.4)
Others	9.85 (8.47 11.23)	8.49 (7.14 9.84)	1.36*** (−0.57 3.29)
**Living arrangement**			
Living alone	20.84 (18.92 22.75)	12.73 (10.07 15.38)	8.11*** (4.83 11.38)
Living with spouse and others	13.21 (12.26 14.15)	14 (13.07 14.93)	−0.79*** (−2.12 0.53)
Living with spouse and children	8.57 (8.15 8.99)	9.35 (8.96 9.75)	−0.79*** (−1.36–0.21)
Living with children and/or others	19.09 (18.32 19.86)	18.66 (17.17 20.15)	0.43*** (−1.25 2.11)
**Smoke**			
No	13.23 (12.87 13.59)	10.93 (10.51 11.36)	2.29*** (1.73 2.85)
Yes	13.01 (11.37 14.66)	11.49 (10.88 12.1)	1.53*** (−0.23 3.28)
**Tobacco**			
No	13.26 (12.87 13.64)	11.23 (10.81 11.64)	2.03*** (1.47 2.6)
Yes	13.01 (12.13 13.88)	10.84 (10.18 11.5)	2.16*** (1.07 3.26)
**Health insurance**			
No	13.54 (13.13 13.94)	11.29 (10.88 11.69)	2.25*** (1.68 2.82)
Yes	12.05 (11.32 12.78)	10.57 (9.87 11.28)	1.48*** (0.46 2.49)
**Comorbidity**			
0	9.33 (8.9 9.75)	7.82 (7.42 8.21)	1.51*** (0.93 2.09)
1	14.26 (13.6 14.93)	12.8 (12.07 13.52)	1.47*** (0.48 2.45)
2+	21.82 (20.85 22.8)	19.81 (18.72 20.9)	2.01*** (0.55 3.48)

**Figure 1:**
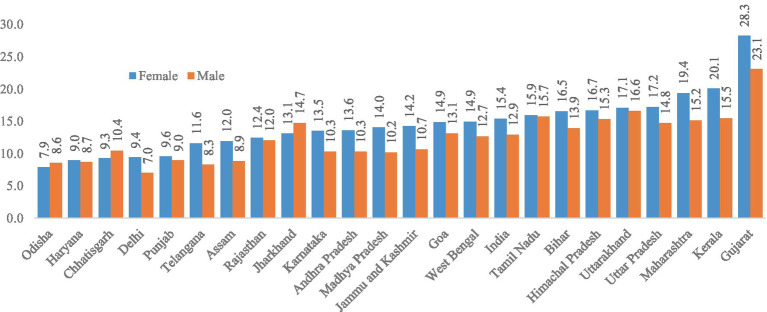
Prevalence of cataracts among older adults across states in India.

Figure 1 shows the prevalence of cataract among older adults across selected states in India. The prevalence of cataract was higher among females than males, except for a few states. The cataract was highest in Gujarat (females 28.31% and males 23.1%), followed by Kerala (females 20.1% and males 15.5%) and Maharashtra (females 19.4% and males 15.2%). The prevalence was lowest in Odisha (females 7.9% and males 8.6%), followed by Haryana (females 9% and males 8.7%) and Chhattisgarh (females 9.3% and males 10.4%).

Table 3 shows the treatment and effective treatment cover of cataract among older adults, segregated by various demographic factors. Notably, 27.52% of older adults in India did not received cataract treatment, showing a significant gap. This gap persists across categories such as gender, with 27.66% of females and 27.34% of males not covered. Furthermore, disparities are evident across age groups, where the 75+ age group has a notably higher percentage of 23.22% not cover for cataract treatment, in contrast to a 35.25% lack of effective treatment coverage in the same group. Economic stratification reveals higher disparities, as the cataract treatment and effective treatment were lowest among the poorest. Around 28.76% of the poorest were not covered for cataract treatment, and those covered, 33.18%, have not received effective treatment. Educational attainment appears to influence coverage, as illiterate individuals show 28.89% not covered and 34.20% lacking effective treatment, while those with 10 or more years of education exhibit lower prevalence. Demographic factors such as residence, caste, religion, and comorbidity also exhibit disparities in the gap.

**Table 3:** Treatment and effective treatment cover of cataract among older adults in India.

Background variables	Cataract treatment cover			Effective cataract treatment cover		
	Yes	No	Total prevalence	Yes	No	Total prevalence
**India**	72.48	27.52	8,149	71.06	28.94	5,883
**Sex**						
Female	72.34	27.66	4,763	69.20	30.80	3,433
Male	72.66	27.34	3,386	73.66	26.34	2,450
**Age group**						
45–54	55.07	44.93	728	79.3	20.70	399
55–64	67.90	32.10	2020	74.04	25.96	1,366
65–74	76.38	23.62	3,318	72.12	27.88	2,524
75+	76.78	23.22	2083	64.75	35.25	1,593
**MPCE quintile**						
Poorest	71.24	28.76	1,685	66.82	33.18	1,196
Poorer	69.61	30.39	1782	68.50	31.50	1,236
Middle	73.56	26.44	1,637	70.94	29.06	1,199
Richer	74.45	25.55	1,665	73.21	26.79	1,235
Richest	74.02	25.98	1,381	76.67	23.33	1,018
**Educational attainment**						
Illiterate	71.11	28.89	4,287	65.80	34.20	3,037
Less than 5 years	75.72	24.28	1,047	73.07	26.93	790
5–9 years completed	72.46	27.54	1,608	74.91	25.09	1,161
10 years or more	74.52	25.48	1,207	82.12	17.88	896
**Residence**						
Rural	71.46	28.54	5,093	68.50	31.50	3,625
Urban	74.17	25.83	3,056	75.16	24.84	2,258
**Caste**						
ST	67.40	32.60	403	68.32	31.68	270
SC	70.04	29.96	1,530	71.33	28.67	1,068
OBC	72.95	27.05	3,706	68.27	31.73	2,693
Others	74.07	25.93	2,509	75.35	24.65	1,851
**Religion**						
Hindu	73.07	26.93	6,712	70.76	29.24	4,885
Muslims	70.36	29.64	984	73.27	26.73	690
Christians	54.01	45.99	209	72.13	27.87	113
Others	80.49	19.51	244	70.10	29.90	196
**Comorbidity**						
0	70.95	29.05	3,335	69.96	30.04	2,357
1	72.16	27.84	2,483	70.45	29.55	1,785
2+	74.99	25.01	2,331	73.17	26.83	1,741

**Figure 2:**
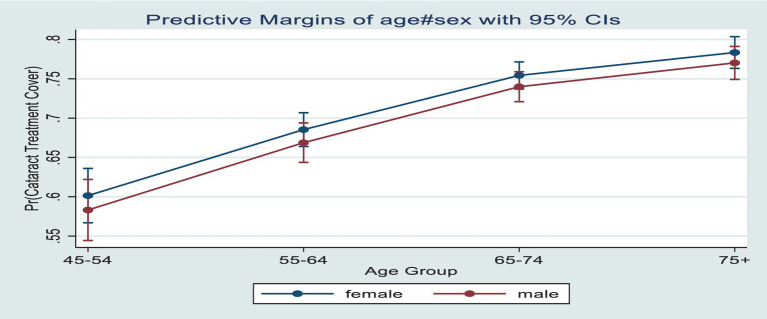
Predictive prevalence of cataract treatment cover by age and sex among older adults in India.

Figure 2 presents the predictive prevalence of cataract treatment covered by age and sex among older adults in India. The predictive prevalence was higher among females compared to males across the age groups, and it increases with age.

**Figure 3:**
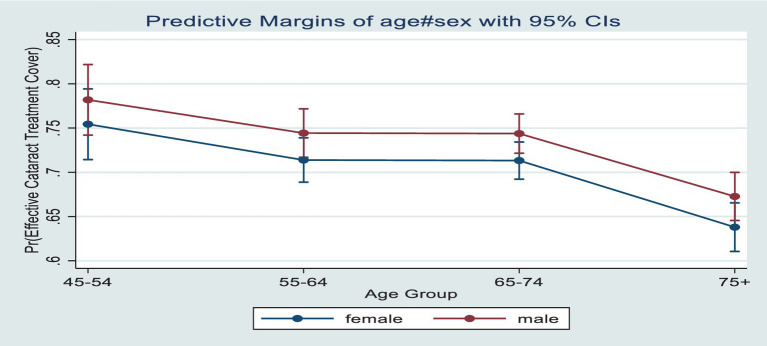
Predictive prevalence of cataract effective treatment cover by age and sex among older adults in India.

Figure 3 presents the predictive prevalence of effective cataract treatment covered by age and sex among older adults in India. In contrast to the cataract treatment cover, the predictive prevalence of effective treatment cover was higher among males compared to females across the age groups. Also, conversely, to the increasing cataract treatment coverage over age, the effective cataract treatment decreases with increasing age.

Table 4 shows the socioeconomic adjusted odds ratio of cataract among older adults. The adjusted odds ratio for cataract was 1.21 [95% CI: 1.08–1.35] times higher among females than males. By MPCE quintile, compared to the richest, the odds of cataract were higher among the poorer 1.12 [95% CI; 0.96–1.31] and poorest 1.07 [95% CI: 0.93–1.25]. The odds of cataract decrease with increasing level of education, and the odds were higher among those educated for less than 5 years 1.28 [95% CI: 1.05–1.57] and illiterates 1.03 [95% CI, 0.87–1.25] compared to those educated for 10 years and more. The odds of cataract increases with the increasing age group; for instance, it was 11.44 [95% CI, 9.42–13.89] times higher among the 75 and above age group than 45–54 age group and around 7.85 [95% CI, 6.59–9.34] times higher among the 55–64 years of age group. Compared to urban residents, the odds were 1.37 [95% CI, 1.22–1.55] times higher in rural areas. By social class, the odds were higher among all except ST; for instance, the odds were 1.78 [95% CI, 1.46–2.15] times higher among the ‘others’ caste than STs. In the case of living arrangements, the odds of cataract were higher among those living alone, 1.35 [1.09–1.68] times compared to those living with a spouse and children. The odds of cataract were higher among those with a chronic condition; for instance, the odds were 1.53 [95% CI, 1.35–1.73] times higher among those who have two and above chronic conditions than those who have none.

**Table 4:** Socioeconomic adjusted odds ratio of cataract among older adults in India.

Background variables	AOR	95% CI
**Sex**		
Male	1	
Female	1.21***	(1.08–1.35)
**Age**		
45–54	1	
55–64	3.43***	(2.91–4.05)
65–74	7.85***	(6.59–9.34)
75+	11.44***	(9.42–13.89)
**MPCE quintile**		
Richest	1	
Richer	1.1*	(0.93–1.31)
Middle	1.06***	(0.91–1.23)
Poorer	1.12***	(0.96–1.31)
Poorest	1.07**	(0.93–1.25)
**Educational attainment**		
10 years or more	1	
5–9 years completed	1.17*	(0.98–1.39)
Less than 5 years	1.28***	(1.05–1.57)
Illiterate	1.03	(0.8 7–1.25)
**Residence**		
Rural	1	
Urban	1.37***	(1.22–1.55)
**Caste**		
Scheduled tribes	1	
Scheduled castes	1.72***	(1.42–2.07)
OBC	1.59***	(1.32–1.91)
Others	1.78***	(1.46–2.15)
**Religion**		
Christian	1	
Hindu	1.08**	(0.70–1.67)
Muslim	1.12***	(0.70–1.78)
Others	0.84**	(0.53–1.36)
**Living arrangement**		
Living with spouse and children	1	
Living alone	1.35***	(1.09–1.68)
Living with spouse and others	1.06	(0.94–1.19)
Living with children and/or others	1.19***	(1.05–1.35)
**Smoke**		
No	1	
Yes	0.97	(0.87–1.09)
**Tobacco**		
No	1	
Yes	0.95	(0.86–1.04)
**Health insurance**		
No	1	
Yes	1.11*	(1.00–1.25)
**Comorbidity**		
0	1	
1	1.21***	(1.09–1.34)
2+	1.53***	(1.35–1.73)

*If *p* < 0.05, **if *p* < 0.01, ***if *p* < 0.001; AOR, adjusted odds ratio; CI, confidence interval.

## Discussion

In India cataract among older adults is a significant public health concern…. This paper examines the prevalence, effective treatment, and treatment disparities among older adults in India.

The prevalence of cataract among older adults in India was 14.25%. This is consistent with previous studies, highlighting cataract as a significant cause of visual impairment and blindness among older adults globally (8, 9). The study also found a higher prevalence of cataract among females than males, which aligns with other studies conducted in India (18, 19). The prevalence of cataract increased with age, with the highest rates observed among the 75 and above age group. Socioeconomic factors were found to be associated with cataract prevalence. The odds of cataract were higher among females, individuals with lower levels of education, and those residing in rural areas. These findings highlight the importance of addressing socioeconomic disparities in cataract treatment and access to healthcare services. Previous studies have also identified income, education, and rural–urban residence as factors influencing the likelihood of cataract surgery (11, 25). Understanding these disparities can help design targeted interventions and improve the accessibility of cataract treatment services for disadvantaged groups. The study also assessed the utilisation of cataract treatment services among older adults. While cataract surgery is highly effective, the findings reveal disparities in both cataract treatment cover and effective treatment cover among older adults in India. While overall, 27.52% of individuals were not covered for cataract treatment, around 28.94% did not received effective treatment, indicating that a significant portion of individuals who undergo treatment do not experience improved vision outcomes. This suggests potential gaps in the quality of healthcare services provided to individuals. Moreover, gender disparities were evident, with a higher proportion of females not exhibiting effective treatment cover (30.80%) compared to males (26.34%). Further, opposite to the cataract treatment cover, the effective treatment cover was higher among male compared to females across the age groups. Conversely the effective cataract treatment decreases with increasing age. Early checkups and treatment may help reduce visual impairment as the chances of surgery outcome coming effective for cataract surgeries will be higher. These findings resonate with a study by Prasad et al. (25), which also identified gender-based discrepancies in cataract treatment outcomes in India. In addition, socioeconomic factors play a pivotal role; individuals with lower educational attainment and belonging to economically disadvantaged groups tend to experience lower effective treatment coverage, reinforcing the influence of socioeconomic status on healthcare access and outcomes. This aligns with global trends; for instance, a study by Lansingh et al. (26) on cataract surgery outcomes across multiple low- and middle-income countries, including India, found that socioeconomic disparities significantly impacted the effectiveness of cataract treatment (26). These findings collectively emphasize the need for targeted interventions that address treatment coverage and quality of care, particularly among vulnerable populations, to bridge the gap in cataract-related vision impairment.

The paper provides valuable insights into the prevalence and risk factors of cataract among older adults in India. However, there are some limitations to consider. The data used in the study relies on self-reported cataract diagnoses Additionally, the LASI survey data is cross-sectional as it was available for only one wave, limiting the ability to establish causal relationships. Future research could benefit from longitudinal studies that track the progression of cataract surgery over time and assess the long-term functional limitations and quality of life outcomes associated with this condition. Cataract treatment disparity in India highlights significant inequalities in access to and quality of healthcare services. Despite being a leading cause of blindness, the availability of effective cataract treatment varies widely across different regions and socio-economic groups. Rural areas and economically disadvantaged populations face substantial barriers, including limited access to specialized medical facilities, lack of trained ophthalmologists, and financial constraints. This disparity has profound implications for policy and practice. Policymakers must prioritize the allocation of resources to under-served areas, enhance training programs for healthcare professionals, and implement subsidized treatment schemes to ensure equitable access to cataract surgery. Addressing these disparities is crucial for reducing preventable blindness and improving overall public health outcomes in India.

In conclusion, cataract pose a significant burden on the ageing population in India. The findings underscore the importance of addressing socioeconomic disparities in access to cataract treatment services and highlight the need for early checkups, early treatment (effective), targeted interventions, and improved healthcare planning. By understanding the epidemiology and implications of cataract, policymakers and healthcare professionals can develop early detection, prevention, and treatment strategies, ultimately improving visual health outcomes and the overall well-being of older adults in India.

## Data availability statement

The original contributions presented in the study are included in the article/supplementary material, further inquiries can be directed to the corresponding author.

## Author contributions

RS: Conceptualization, Data curation, Formal analysis, Investigation, Methodology, Resources, Software, Visualization, Writing – original draft, Writing – review & editing. SM: Supervision, Writing – review & editing.
